# The entanglement between flaviviruses and ER-shaping proteins

**DOI:** 10.1371/journal.ppat.1008389

**Published:** 2020-04-16

**Authors:** Maaran Michael Rajah, Blandine Monel, Olivier Schwartz

**Affiliations:** 1 Institut Pasteur, Virus and Immunity Unit, CNRS-UMR3569, Paris, France; 2 Ecole Doctorale Bio Sorbonne Paris Cité (BioSPC) -Université de Paris, Paris, France; 3 Vaccine Research Institute, Creteil, France; Mount Sinai School of Medicine, UNITED STATES

## Love at first site: The endoplasmic reticulum and Flavivirus replication

The endoplasmic reticulum (ER) is a continuous intracellular membrane system that composes the nuclear envelope and radiates out to the peripheries of the cell, transitioning from ribosome-studded, flat cisternal sheets to highly curved reticulated tubules. It has been implicated in a diverse array of cellular functions, including production, trafficking, and degradation of proteins; synthesis and distribution of lipids and steroids; calcium sequestration and release; cell signaling and innate immunity; carbohydrate metabolism; and detoxification of harmful substances [1]. Such a ubiquitous and functionally versatile organelle is parasitized by viruses in order to facilitate their life cycle. Rotavirus, vaccinia virus (VV), hepatitis C virus (HCV), as well as members of the Flavivirus genus—dengue virus (DENV), West Nile virus(WNV), yellow fever virus (YFV), and Zika virus (ZIKV)—are all intimately associated with the ER [2]. The flaviviruses replicate on the ER membranes and form immature viral particles that bud into the ER lumen. These particles are then trafficked through the ER–Golgi network and undergo a maturation process brought about by pH alteration and cleavage by the host-protease Furin. This viral takeover of the ER requires the co-opting of cellular proteins as well as the active remodeling of the ER membrane to create a cellular environment more conducive to replication [3]. Several ER proteins have been identified as host factors in flavivirus replication [4], but a class of resident proteins that shape and maintain the dynamic ER architecture are of particular interest. These “ER-shaping” proteins could potentially serve as host factors that support viral replication or restriction factors that protect the ER. Interactome and CRISPR screens have produced somewhat discordant results, but the less stringent screens suggest that ER-shaping proteins may be targets of viral proteins [5] [6] [7]. This Pearl review will explore the virus-induced ER-membrane rearrangement and the relationship between ER-shaping proteins and the flavivirus life cycle.

## Virulent and manipulative passions: Virus-induced manipulation to the ER structure

**Fig 1:**
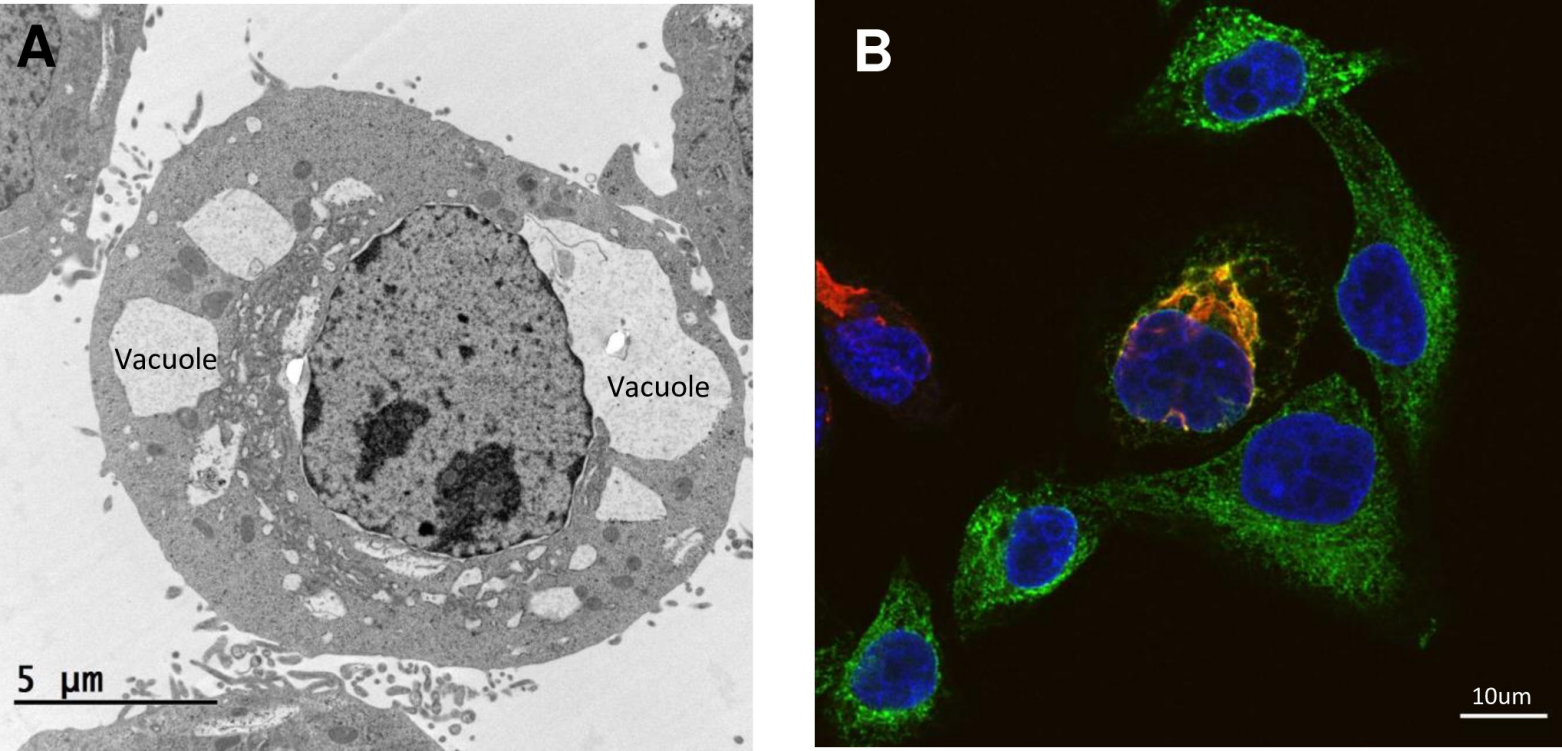
Microscopy images of ZIKV-infected HeLa Cells 24 hours after infection. (A) Electron micrograph of ER-derived vacuoles in a ZIKV-infected cell. (B) Confocal microscopy image showing the colocalization (yellow/orange) of ER-shaping protein ATL3 (green) with the ZIKV protein NS3 (red). ATL3, Atlastin-3; ER, endoplasmic reticulum; NS3, nonstructural protein 3; ZIKV, Zika virus.

Virus-induced morphological changes to the ER membrane are brought about by active restructuring facilitated by interactions between viral proteins and host factors. The functional benefits of membrane alterations include the spatial compartmentalization of the viral-replication machinery—increasing the accessibility and concentration of necessary host factors—protection from the innate immune response, and the possibility of increased viral spread [3] [8]. The characteristics of the replication-supporting ER-membrane structures differ between viruses. HCV forms a “membranous web” and double-membrane vesicles, and VV manipulates the ER into wrapping around the cytosolic site of viral-DNA replication [9] [10]. Flaviviruses form invaginations in the ER referred to as vesicle packets (VPs), which are clusters of vesicle membranes housing the viral-replication machinery [3] [11] [12] [13] [14]. Immature flavivirus virions form paracrystalline arrays within the ER lumen [11]. In some cell types, flaviviruses also form convoluted membranes (CMs), which are potential sites for the translation and processing of viral proteins [3] [11] [12]. Interestingly, ZIKV infection in certain cell types produces large ER-derived vacuoles, which are followed by an implosive cell death (Fig 1A) [8]. Several Flavivirus proteins and host factors (i.e., reticulophagy factor FAM134B [15]) have been implicated in the ER-modification process. Of recent interest are ER-shaping proteins, that can be operationally subdivided into proteins that help form the structure and curvature of the ER and fusogens that maintain the reticulated ER network.

## Curvy and sturdy: ER curvature-stabilizing proteins and virus interactions

The class of ER-shaping proteins labeled as curvature-stabilizing proteins are subdivided into two families, the receptor expression-enhancing protein (REEP)/DP1/yop1p and the reticulons [1]. The REEPs are a family of six proteins that interact with microtubules through an extended C-terminal cytoplasmic domain. By facilitating the association of the ER membrane to cytoskeletal dynamics, they provide the mechanics necessary to extend ER tubules, as well as to form the positive curvature of the ER membrane [16] [1]. Mutations in the REEP1 proteins are associated with hereditary spastic paraplegias (HSP), a family of inherited neurological disorders characterized by spastic weakness in the extremities, which is partly reminiscent of symptoms induced by neurotropic flaviviruses [1] [16]. The effect of the REEP family on flavivirus replication has not been thoroughly explored, with one investigation reporting that the depletion of REEP1 does not affect DENV replication [17].

The reticulon family consists of four (Reticulon 1 [RTN1] to RTN4) membrane-bound proteins that contain hydrophobic domains occupying the outer leaflet of the ER membrane. The resultant hydrophobic wedging, and possibly the scaffolding and protein–protein crowding formed by reticulon oligomers, contributes to the curvature of the ER membrane [1]. Several studies have identified reticulons as host factors in viral replication; Enterovirus 71 protein 2C and brome mosaic virus protein 1a both interact with RTNs to facilitate replication [18] [19]. Contrarily, RTN3 acts as a restriction factor in HCV replication by preventing nonstructural protein 4A (NS4A) self-interaction [20].

**Fig 2:**
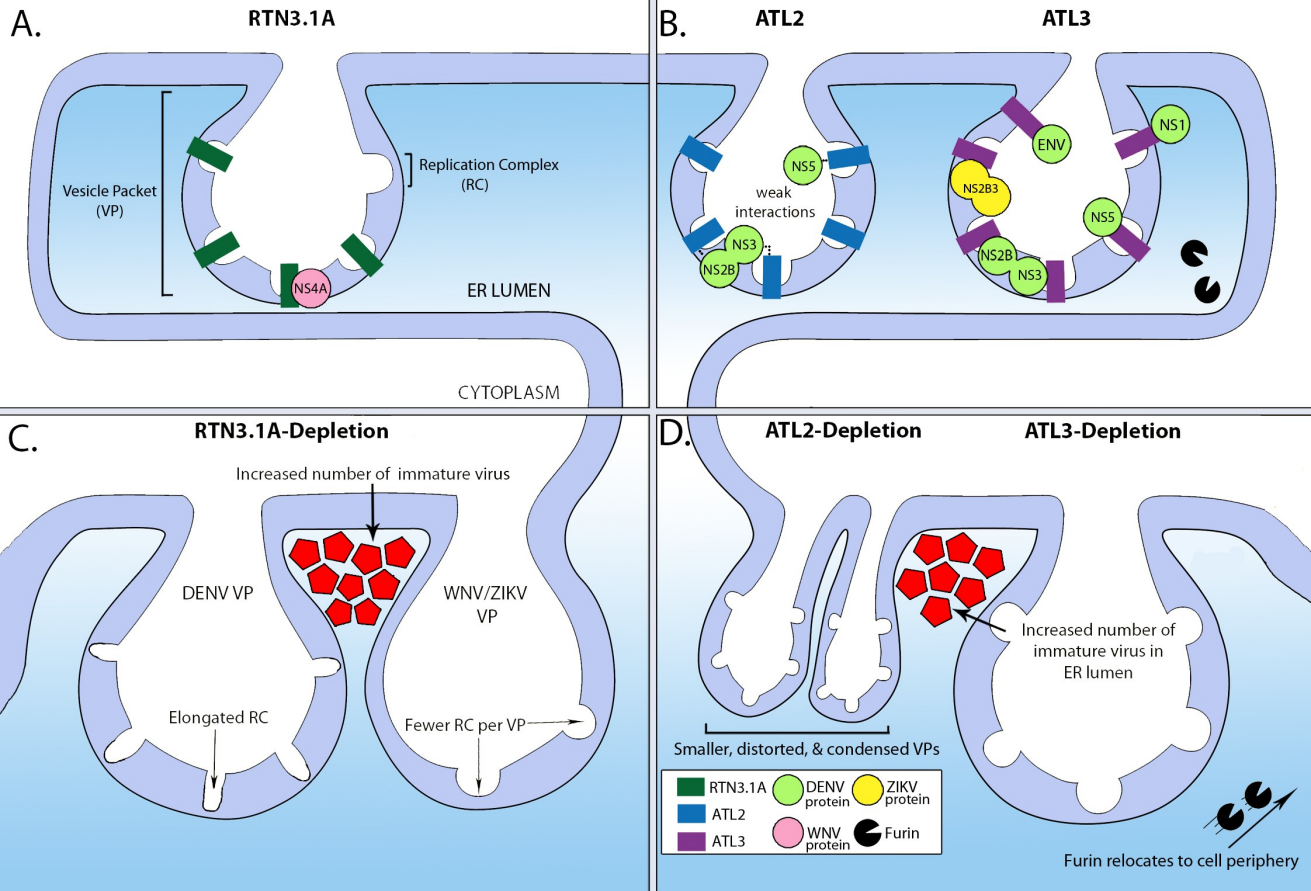
The relationship between ER-shaping proteins and flaviviruses during infection. A simplified schematic showing the interaction between different flavivirus proteins and ER-shaping proteins: (A) RTN3.1A and (B) ATL2 (left) and ATL3 (right). (C) Depleting RTN3.1 results in elongated RCs in DENV-infected cells and fewer RCs per VP in WNV-infected and ZIKV-infected cells. (D) Depleting ATL2 (left) results in VPs that are smaller, distorted, and condensed but does not change the overall number of VPs, whereas ATL3 depletion results in the host-protease Furin relocating from the perinuclear region to the cell periphery and accumulation of immature virus particles in the ER lumen. Immature virus particles are represented smaller than they would be in relation to VPs. ATL2, Atlastin-2; ATL3, Atlastin-3; DENV, dengue virus; ENV, envelope; ER, endoplasmic reticulum; NS1, nonstructural protein 1; RC, replication complex; RTN3.1A, Reticulon 3.1A; VP, vesicle packet; WNV, West Nile virus; ZIKV, Zika virus.

DENV, WNV, and ZIKV recruit RTN3.1A to the viral-replication site [21]. RTN3.1A depletion reduces viral titers and decreases viral RNA and protein levels [21]. The RTN3.1A colocalizes with the NS4A proteins of DENV, ZIKV, and WNV, but it only directly interacts with WNV NS4A via its N-terminal transmembrane domain (Fig 2A) [21]. The expression of WNV and DENV NS4A alone is sufficient to induce ER rearrangements [22] [23]. Depletion of RTN3.1A differentially alters the formation of the ER-derived replication organelles between flaviviruses. In ZIKV-infected and WNV-infected cells, there is a noticeable decrease in the amount of vesicle membranes present per VP (Fig 2C) [21]. While amount of vesicle membranes per VP was not altered in DENV-infected cells, their morphology became more elongated (Fig 2C), and there was an increase in immature viral particles upon RTN3.1A depletion (Fig 2C) [21]. Overall, while RTN3.1A may differently influence the life cycle of specific flaviviruses, it interacts with viral proteins and induces morphological changes to the ER in order to promote replication.

## Together at-last-in each other’s embrace: Virus interaction with the atlastin fusogens

The atlastins (ATLs) are a family of three membrane-bound, dynamin-related guanosine triphosphate (GTP)ases that mediate the construction of the reticulated ER network. The hydrolysis of GTP dissociates *cis*-membrane dimers and supports the tethering and dimerization with ATLs situated on a different ER membrane [24]. The homotypic fusion of ER membranes results in the formation of three-way junctions, shaping the smooth ER into an intricate and dynamic web of tubules. The depletion of ATL reduces the density of the ER network [25], and mutations in ATLs are also associated with HSP. In addition to their role as an ER-shaping protein, ATLs also influence protein targeting to the inner nuclear membrane and the biogenesis of nuclear pore complexes, regulate lipid droplet size, and facilitate selective autophagy [26] [27] [28]. Thus, ATLs could potentially be expedient host factors for several pathogens. A previous study implicated ATL3 in remodeling the ER to promote the formation of vacuoles that facilitate the intracellular replication of *Legionella pneumophila* [29].

Two recent investigations examined the relationship between the three ATLs and different flaviviruses and found varying effects based on the virus [17] [30]. Silencing ATL2 resulted in a significant reduction of DENV, WNV, and ZIKV titers and viral RNA [17]. ATL2 depletion affected formation of ER-derived DENV-replication organelles, distorting the size and shape of the vesicles and condensing them into a small perinuclear region (Fig 2D) [17]. The depletion of ATL3 reduced the titers of ZIKV and DENV (but not WNV) and had no effect on viral-RNA levels [17] [30]. ATL3 depletion did not change the morphology of the DENV-induced vesicles but resulted in an increased accumulation of intracellular viral particles in the ER lumen (Fig 2D) [17]. The GTPase function of ATLs is important for DENV and ZIKV replication [30] [17].

The different ATL proteins exhibit variation in their interaction with flavivirus proteins. ATL3 relocates to the ZIKV-replication site and directly interacts with nonstructural protein 3 (NS3; Fig 1B) and NS2B3, that belong to the viral-replication complex (Fig 2B) [30]. Both ATL2 and ATL3 interact with DENV NS3, NS5, and NS2B proteins, though interactions with ATL2 are relatively weak (Fig 2B) [17]. DENV NS1 protein and the envelope and capsid proteins also interact with ATL3 (Fig 2B) [17]. ATL3 is recruited to DENV-replication organelles and is enriched in the membrane-surrounding virions, but it is not necessarily associated with the site of viral-RNA replication [17]. In DENV-infected cells, ATL3 interacts with ADP-ribosylation factor 4 (ARF4), a protein that is associated with trafficking of proteins and vesicle processing [17]. Depletion of AFR4 and the related ARF5 protein impairs DENV assembly and release. The depletion of ATL3 results in the relocalization of Furin from the perinuclear region to the cell periphery (Fig 2D), suggesting that ATL3 may play a role in providing immature viral particles access to Furin (Fig 2D) in addition to a potential direct role on assembly and trafficking of viral particles [17]. Overall, these studies show that ATL3 is associated with the cytoplasmic transport of vesicles and is intimately involved with flavivirus assembly and maturation [17].

## Happily, ever after: How the future could shape out

ER-shaping proteins impact the life cycle of flaviviruses by interacting with viral proteins, influencing viral assembly and maturation and promoting the formation of ER-derived factories. Future investigations could examine the role of other ER-shaping proteins including Spastin, Lunapark, and other REEP proteins that are involved in the regulation of ER shape through microtubule dynamics, stabilization of three-way ER tubular junctions, and formation and stabilization of the ER curvature, respectively. It will be worth investigating if ER-shaping proteins are involved in ZIKV-induced cytoskeleton modifications [12] and the formation of vacuoles to understand if these proteins are related to ER stress, innate sensing of infection, and viral replication [30] [8] [29]. The interplay between different ER-shaping proteins [31] within the context of viral infection is also of potential interest. Finally, since mutations in several of the ER-shaping proteins are associated with inherited neurological complications, it would be interesting to determine if they are implicated in the pathology induced by neurotropic flaviviruses in relevant cell and animal models [32].
